# New Cassane Diterpenoids from *Caesalpinia sappan* and Their Antiplasmodial Activity

**DOI:** 10.3390/molecules22101751

**Published:** 2017-10-17

**Authors:** Nai-Liang Zhu, Zhong-Hao Sun, Mei-Geng Hu, Tong-Yu Wu, Jing-Quan Yuan, Hai-Feng Wu, Yu Tian, Peng-Fei Li, Jun-Shan Yang, Guo-Xu Ma, Xu-Dong Xu

**Affiliations:** 1 Key Laboratory of Bioactive Substances and Resource Utilization of Chinese Herbal Medicine, Ministry of Education, Beijing Key Laboratory of Innovative Drug Discovery of Traditional Chinese Medicine (Natural Medicine) and Translational Medicine, Key Laboratory of Efficacy Evaluation of Chinese Medicine against Glycolipid Metabolic Disorders, State Administration of Traditional Chinese Medicine, Institute of Medicinal Plant Development, Peking Union Medical College and Chinese Academy of Medical Sciences, Beijing 100193, China; zhu13liang@126.com (N.-L.Z.); sun_zhonghao@126.com (Z.-H.S.); Humeigeng@126.com (M.-G.H.); hfwu@implad.ac.cn (H.-F.W.); ytian@implad.ac.cn (Y.T.); lipengfei1121@126.com (P.-F.L.); jsyang@implad.ac.cn (J.-S.Y.); 2 Center of Research and Development on Life Sciences and Environment Sciences, Harbin University of Commerce, Harbin 150076, China; wty_lostheaven@163.com; 3 College of Chemistry and Materials Science, Guangxi Teachers Education University, Nanning 530001, China; yjqgx@163.com

**Keywords:** *Caesalpinia sappan*, cassane diterpenes, *N* bridge, antiplasmodial activity

## Abstract

One new cassane diterpene possessing an unusual *N* bridge between C-19 and C-20 named caesalsappanin R (**1**), as well as another new diterpene caesalsappanin S (**2**), were isolated from the seeds of *Caesalpinia sappan* with methanol extract. Their structures were determined by spectroscopic analysis and examined alongside existing data from prior studies. Their biological activities were profiled by their antiplasmodial activity.

## 1. Introduction

**Figure 1:**
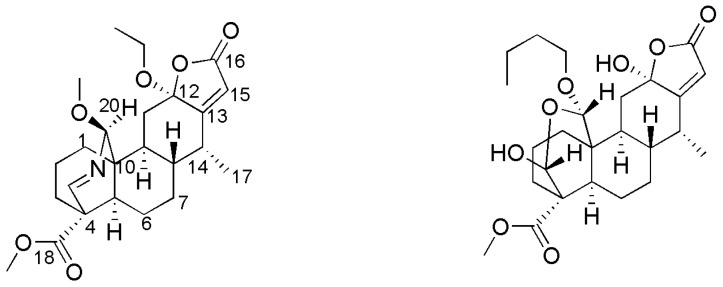
The structures of compounds **1**–**2**.

*Caesalpinia* *sappan* has been a part of traditional Chinese herbal medicine and is widely used in the treatment of dysmenorrheal, blood stagnation, and tetanus. Previous phytochemical investigations indicated that this genus contains an abundant source of cassane diterpenes with different structure types, and most of them showed in vitro or in vivo pharmacological impacts such as antiproliferative [1,2,3], antiplasmodial [4,5], antibacterial [6], antihelmintic, and antineoplastic activity [7]. As a continuation of our project towards new bioactive diterpenes discovery from the genus *Caesalpinia* [2,3,8], we examined the chemical constituents of *C*. *sappan* and obtained two new cassane diterpenes, designated caesalsappanin R (**1**) and caesalsappanin S (**2**) (Figure 1). Compound **1** is a rather unusual cassane diterpenoid lactone-type skeleton, consisting of an *N* bridge between C-19 and C-20. In this paper, we detail the separation and structural determination of the novel agents and the examination of their antiplasmodial activity.

## 2. Results and Discussion

### 2.1. Purification of Compounds ***1**–**2***

The seeds of *C. sappan* were extracted with MeOH three times. The two cassane-type diterpenoids were isolated and purified via silica gel chromatography, Sephadex LH-20 gel chromatography and semi-HPLC.

### 2.2. Structure Elucidation of Compounds ***1**–**2***

**Figure 2:**
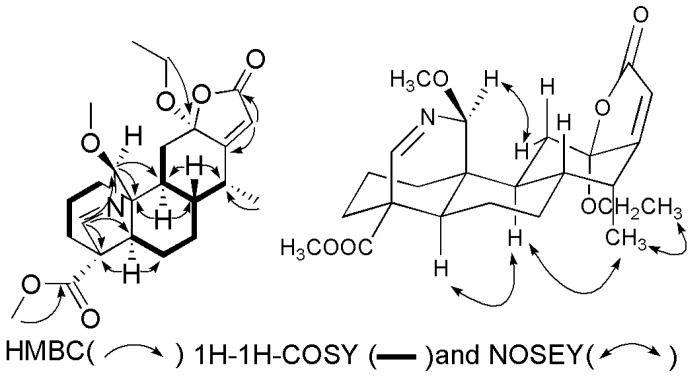
Key 2D NMR correlations of compound **1**.

Compound **1** was acquired as a white shapeless powder. The HRESIMS spectrum demonstrated a quasi-molecular ion at *m*/*z* 454.2199 (Calcd. for C_24_H_33_NO_6_Na, 454.2206), which in connection with the NMR data, confirmed that the molecular formula was C_24_H_33_NO_6_. The IR and UV spectra revealed absorptions for an amidogen (3190 cm^−1^), a carbonyl (1735 cm^−1^), and an *α*,*β*-unsaturated butenolide unit (210 nm; 1749 cm^−1^) [2]. The ^1^H and ^13^C APT NMR spectra (Table 1) displayed the olefinic proton signal at *δ*_H_ 5.86 (H-15, s) and four downfield-shifted carbon signals at *δ*_C_ 107.4 (C-12), 171.0 (C-13), 115.9 (C-15), and 179.9 (C-16), which also confirmed the presence of the *α*,*β*-unsaturated butenolide ring. Additionally, the ^1^H-NMR spectrum exhibited signals for a methyl at *δ*_H_ 1.14 (d, *J* = 7.2 Hz, H_3_-17), two methoxys at *δ*_H_ 3.74 (s, 18-OCH_3_) and 3.72 (s, 20-OCH_3_), an ethoxy group at *δ*_H_ 3.30, 3.58 (m, OCH_2_CH_3_) and 1.21 (d, *J* = 7.2 Hz, OCH_2_CH_3_), a nitrogen oxymethylene proton at *δ*_H_ 5.07 (d, *J* = 2.4 Hz), and a nitrogen alkenyl at *δ*_H_ 7.53 (s). Except for the methoxy (*δ*_C_ 52.2, 57.1) and ethoxy (*δ*_C_ 59.3, 15.0) substituents, the ^13^C APT NMR spectrum showed 20 carbons including one methyl (*δ*_C_ 12.2), six methylenes (*δ*_C_ 19.3, 25.6, 29.1, 30.2, 33.1, and 37.2), seven methines (*δ*_C_ 37.0, 42.2, 43.4, 47.0, 91.2, 115.9, and 169.9), and six quaternary carbons (*δ*_C_ 44.1, 49.8, 107.4, 169.9, 171.0, and 175.3). The HSQC spectrum displayed all of the proton signals assigned to the corresponding carbons through direct ^1^H and ^13^C correlations. The overall ^1^H- and ^13^C-NMR spectroscopic data confirmed that **1** is an oxynitride diterpene possessing a fused butenolide unit [9,10], and its entire structure was connected, as confirmed using HSQC, HMBC, and ^1^H-^1^H-COSY spectra (Figure 2). The nitrogen oxymethylene proton at *δ*_H_ 5.07 (d, *J* = 2.4 Hz, H-20) showed long-range correlations with carbons at *δ*_C_ 30.2 (C-1), 49.8 (C-10), 162.6 (C-19), and 57.1 (20-OCH_3_), which suggested that C-1, C-10, C-19, and −OCH_3_ were connected through the nitrogen oxymethylene carbon C-20. The quaternary carbon C-4 (*δ*_C_ 44.1) was connected to C-3 (*δ*_C_ 33.1), C-5 (*δ*_C_ 47.0), C-18 (*δ*_C_ 175.3), and C-19 (*δ*_C_ 162.6) due to the HMBC correlations of H-19, H_2_-3, and H-5 to C-4 and C-18. Moreover, the nitrogen bridge between C-19 and C-20 was confirmed by the downfield chemical shifts of C-19 (*δ*_C_ 162.6) and C-20 (*δ*_C_ 91.2) together with the HMBC correlations between H-19 and C-20. Finally, the *α,β*-unsaturated butenolide moiety was connected to C-11 and C-14 based on the HMBC correlations from H_2_-11 to C-12 (*δ*_C_ 107.4) and H-14 to C-13 (*δ*_C_ 171.0). The proton H_3_-17 (*δ*_H_ 1.14, d, *J* = 7.2 Hz) showed long-range correlations with carbons C-14 (*δ*_C_ 37.0), which indicated that the methyl group of C-17 was connected to C-14. The methoxyl and ethoxyl groups were attached to C-18 and C-12, respectively, based on the HMBC correlations between *δ*_H_ 3.74 (s, −OCH_3_) and *δ*_C_ 175.3 (C-18), *δ*_H_ 3.30, 3.58 (m, OCH_2_CH_3_) and *δ*_C_ 107.4 (C-12). The NOESY experiment established the relative configuration of compound **1**, the correlations of H-20 (*δ*_H_ 5.07)/H-1*α* (*δ*_H_ 1.69–1.72), H_3_-17 (*δ*_H_ 1.14)/H-9 (*δ*_H_ 1.78), OCH_2_CH_3_-12 (*δ*_H_ 3.30)/H_3_-17 (*δ*_H_ 1.14) showed that the hydroxyl group was *β*-oriented at C-20, and the methyl group at C-14 and the ethoxy group at C-12 were all *α*-oriented. The same carbon skeleton with the *trans/anti/trans* system of three six-membered rings A, B, and C, and the oriented proton at C-8 was *β*-axial and the oriented protons at C-5/C-9 was *α*-axial, which are well established on all cassane diterpenes isolated so far from the genus *Caesalpinia* [3,8,11]. Considering the biosynthetic relationship and comparing with the literature of cassane diterpenoids [12], the absolute configurations of the chiral carbons were determined to be 4*S*, 5*R*, 8*S*, 9*S*, 10*S*, 12*S*, 14*R* in **1** and are shown in Figure 2. Therefore, the structure of **1** was determined and it was named caesalsappanin R (Figure 1). Compound **1** is representative of a new cassane diterpenoid lactone-type skeleton with an *N* bridge between C-19 and C-20.

Compound **2** was separated as a white shapeless powder, [α]${}_{D}^{20}$ −47.3 (*c* = 0.1, MeOH). Its molecular formula was determined to be C_25_H_36_O_8_ by HRESIMS (observed *m*/*z* 487.2332 [M + Na]^+^). The ^1^H- and ^13^C-NMR data displayed a cassane diterpene skeleton with an oxygen bridge between C-19 and C-20, which was very similar to the reported compound caesalsappanin H [3]. In fact, the only difference between them was that the methoxy group at C-20 in caesalsappanin H was replaced with a butoxy group in **2**. The ^1^H- and ^13^C-NMR spectra displayed the signals at *δ*_H_ 3.22, 3.80 (2H, ddd, *J* = 9.6, 6.0, 3.0 Hz, CH_2_), 1.32, 1.47 (2H, m, CH_2_), 1.28, 1.43 (2H, m, CH_2_), 1.87 (3H, t, *J* = 7.2 Hz, CH_3_) and *δ*_C_ 67.8 (CH_2_), 31.9 (CH_2_), 20.0 (CH_2_), 13.7 (CH_3_), which suggested the presence of a butoxy group. Also, the HMBC correlations from CH_2_ (*δ*_H_ 3.22, 3.80) to C-20 (*δ*_C_ 104.2) supported the position of the butoxy group at C-20. Taken along with ^1^H-^1^H COSY, HSQC, HMBC, and NOE spectra, the structure of compound **2** was determined and it was named caesalsappanin S (Figure 1).

### 2.3. In Vitro Antiplasmodial and Larvicidal Activities of Compounds ***1**–**2***

The two compounds were tested against the chloroquine-resistant strain K1 of *P. falciparum* (Table 2). Compound **1** exhibited relatively good antiplasmodial activity in vitro with IC_50_ values of 3.6 μM, compared with chloroquine. On the other hand, compound **2** showed only weak activity against the chloroquine-resistant K1 strain of *P. falciparum*. It appears that the presence of the *N* bridge in cassane-type diterpenoids may play an important role in enhancing activity against the chloroquine-resistant K1 strain of *P. falciparum* in vitro. Furthermore the toxic activity of compounds **1** and **2** against mosquito larvae was evaluated. Both compounds displayed only low activity.

## 3. Materials and Methods

### 3.1. General Experimental Procedures

Optical rotation data were measured with a Perkin-Elmer 341 digital polarimeter (PerkinElmer, Norwalk, CT, USA). UV and IR data spectra were recorded on Shimadzu UV2550 and FTIR-8400S spectrometers (Shimadzu, Kyoto, Japan). NMR spectra were obtained using a Bruker AV III 600 NMR spectrometer with chemical shift values presented as *δ* values having TMS (Tetramethylsilane) as the internal standard. HRESIMS was performed using an LTQ-Orbitrap XL spectrometer (Thermo Fisher Scientific, Boston, MA, USA). Column–chromatography (CC) was performed using silica gel (100–200 and 300–400 mesh, Qingdao Marine Chemical Plant, Qingdao, China), Sephadex LH-20 (Pharmacia, Uppsala, Sweden). Precoated silica gel GF_254_ plates (Zhi Fu Huang Wu Pilot Plant of Silica Gel Development, Yantai, China) were used for TLC. All solvent used was of analytical grade (Beijing Chemical Plant, Beijing, China).

### 3.2. Plant Material

The seeds of *C. sappan* were collected from Nanning, Guangxi Province, People’s Republic of China, in April 2013, and identified by Professor Jing Quan Yuan of the Department of Pharmaceutical Chemistry, Guangxi Botanical Garden of Medicinal Plants. A voucher specimen (NO. 13418) was deposited at the Guangxi Botanical Garden of Medical Plants.

### 3.3. Isolation and Purification of Compounds ***1**–**2***

The air-dried seeds of *C*. *sappan* (5.0 kg) were extracted with MeOH (3 × 40 L, 3 h each) at room temperature. Removal of the MeOH under reduced pressure yielded a MeOH-soluble extract (1267 g). The residue was suspended to H_2_O (3 L) and partitioned with petroleum ether (3 × 3 L), CH_2_Cl_2_ (3 × 3 L), EtOAc (3 × 3 L), and n-BuOH (3 × 3 L), successively. The EtOAc fraction (164 g) was subjected to CC (column–chromatography) over silica gel (100–200 mesh, 15 × 60 cm) eluting with a stepwise gradient of CH_2_Cl_2_–MeOH (from 1:0 to 0:1, 100:0, 90:1, 70:1, 50:1, 30:1, 20:1, 10:1, 5:1, 2:1, 1:1, 0:1, *v*/*v*) to afford fractions A–G. Fraction E (3.1 g) was subjected to chromatographed repeatedly over silica gel CC eluting with CH_2_Cl_2_–MeOH (50:0, 40:1, 30:1, 20:1, 10:1, *v*/*v*) to obtained sub-fractions Fractions E1–E5. Fraction E3 was separated using silica gel CC eluting with CH_2_Cl_2_–MeOH (40:1, 30:1, 0:100, *v*/*v*) to obtained sub-fractions I-III. Sub-fraction II was purified by semi-HPLC of MeOH–H_2_O (55:45, *v*/*v*) as the mobile phase to yield compound **1** (6.3 mg, 0.000146%, *t*_R_ = 28.4 min). Fraction E2 was separated using silica gel CC eluting with CH_2_Cl_2_–MeOH (50:1, *v*/*v*), yielding compound **2** (8.6 mg, 0.000172%).

### 3.4. Characterization of Compounds ***1**–**2***

Caesalsappanin R (**1**), White powder (MeOH); [α]${}_{D}^{20}$ −24.2 (*c* = 0.05, MeOH); UV (MeOH) λ_max_ (log ε) 210 (3.86) nm; IR (film) ν_max_ 3190, 1745, 1735 cm^−1^; ^1^H- and ^13^C-NMR data (CDCl_3_), see (Table 1); HR-ESI-MS *m*/*z* 454.2199 [M + Na]^+^. (Calcd. for. 454.2206 C_24_H_33_NO_6_Na).

Caesalsappanin S (**2**), White powder (MeOH); [α]${}_{D}^{20}$ −47.3 (*c* = 0.1, MeOH); UV (MeOH) λ_max_ (log ε) 213 (3.94) nm; IR (film) ν_max_ 3450, 1730 cm^−1^; ^1^H- and ^13^C-NMR data (CDCl_3_), see (Table 1); HR-ESI-MS *m*/*z* 487.2332 [M + Na]^+^. (Calcd. for. 487.2308 C_24_H_34_O_8_Na).

### 3.5. Antiplasmodial Assays of Compounds ***1**–**2***

Antiplasmodial activity in vitro was determined by means of the microculture radioisotope technique based on the method described by Desjardins [13]. The parasite *P. falciparum* (K1, multidrug-resistant strain) was cultured continuously according to the method of Trager and Jensen [14]. Three preparations were used for each experiment. The determination of IC_50_ values against the erythrocytic stages of *P. falciparum* was carried out in duplicate using the [3*H*]-hypoxanthine incorporation assay [15]. Laboratory colonies of mosquito larvae/pupae (*Culex quinquefasciatus* Say, Diptera, Culicidae) were used for the larvicidal/pupicidal activity. Twenty-five numbers of first to fourth instars larvae and puape were introduce into 500 mL glass beaker containing 249 mL of de-chlorinated water and 1 mL of desired concentrations of ethanolic leaf extract were added. Larval food was given for the test larvae. At each tested concentration two to five trials were made and each trial consisted of five replicates. The control was setup by mixing 1 mL of acetone with 249 mL of dechlorinated water. The larvae and pupae were exposed to dechlorinated water without acetone served as control. The control mortalities were corrected by using Abbott’s formula [16,17]. The LC_50_ were calculated from toxicity data by using probit analysis [18]. Chloroquine was included as a standard for comparison. Data are presented as means ± SEM. Statistical analyses were done by means of the Student’s *t*-test. A *P* value of less than 0.05 was considered a significant difference.

## 4. Conclusions

In conclusion, two new cassane-type diterpenoids (**1** and **2**) were isolated and characterized by spectrometric analysis (1 and 2D NMR, HRESIMS). Compound **1** exhibited active antiplasmodial activity in vitro with IC_50_ at 3.60 μM. In addition, the compounds that we had reported also showed antiplasmodial activities; caesalsappanins A, G, H, and I displayed antiplasmodial activities with IC_50_ values of 7.4, 0.78, 0.52, and 2.5 μM, respectively [3]. Therefore, we believe that this plant is an important source for the diverse structure of cassane-type diterpenoids and should be further investigated for the antiplasmodial activity.

## Figures and Tables

**Table 1:** NMR spectral data of **1**–**2** (CDCl_3_, 600 and 150 MHz).

No.	1		2		Caesalsappanin H
	*δ*_C_, Type	*δ*_H_ (*J* in Hz)	*δ*_C_, Type	*δ*_H_ (*J* in Hz)	*δ*_C_, Type
1	30.2 CH_2_	1.69–1.72 (m)	37.7 CH_2_	1.28–1.30 (m)	37.8 CH_2_
		2.17–2.21 (m)		2.07–2.09 (m)	
2	19.3 CH_2_	1.38–1.41 (m)	20.6 CH_2_	1.58–1.60 (m)	20.6 CH_2_
		2.59–2.63 (m)		2.23–2.25 (m)	
3	33.1 CH_2_	1.28–1.32 (m)	28.5 CH_2_	1.82–1.83 (m)	28.6 CH_2_
		1.89–1.93 (m)		2.25–2.30 (m)	
4	44.1 C		50.3 C		50.4 C
5	47.0 CH	1.73 (m)	47.2 CH	1.68–1.71 (m)	47.2 CH
6	25.6 CH_2_	1.18–1.20 (m)	24.2 CH_2_	1.19–1.21 (m)	24.2 CH_2_
		1.39–1.42 (m)		2.00–2.02 (m)	
7	29.1 CH_2_	1.69–1.72 (m)	29.5 CH_2_	1.25–1.28(m)	29.5 CH_2_
		2.19–2.23 (m)		1.60–1.62 (m)	
8	43.4 CH	1.49 (m)	41.5 CH	2.19–2.21 (m)	41.1 CH
9	42.2 CH	1.78 (m)	41.3 CH	1.51–1.53 (m)	41.3 CH
10	49.8 C		38.6 C		38.7 C
11	37.2 CH_2_	1.68–1.70 (m)	38.0 CH_2_	1.36–1.38 (m)	38.1 CH_2_
		2.75 (dd, 12.0,2.4)		2.51–2.53 (m)	
12	107.4 C		105.5 C		105.9 C
13	171.0 C		173.4 C		173.7 C
14	37.0 CH	2.99 (qd, 7.2, 2.4)	37.1 CH	2.91 (qd, 7.2, 2.4)	37.1 CH
15	115.9 CH	5.86 (s)	113.5 CH	5.69 (s)	113.8 CH
16	169.9 C		170.7 C		170.7 C
17	12.2 CH_3_	1.14 (d, 7.2)	12.0 CH_3_	1.13 (d, 7.2)	12.1 CH_3_
18	175.3 C		175.6 C		175.5 C
19	162.6 CH	7.53, s	90.1 CH	5.60 (s)	90.1 CH
20	91.2 CH	5.07 (d, 2.4)	104.2 CH	4.49 (s)	105.4 CH
	59.3, CH_2_	3.30 (m)			
		3.58 (m)			
OCH_2_CH_3_-12	15.0, CH_3_	1.21 (t, 7.2)			
OCH_3_-18	52.2	3.74 (s)	52.0	3.71 (s)	51.7
OCH_3_-20	57.1	3.72 (s)			55.7
OCH_2_CH_2_CH_2_CH_3_-20			67.8	3.22 (ddd, 9.6, 6.0, 3.0) 3.80 (ddd, 9.6, 6.0, 3.0)	
OCH_2_CH_2_CH_2_CH_3_-20			31.9	1.32 (m)	
				1.47 (m)	
OCH_2_CH_2_CH_2_CH_3_-20			20.0	1.28 (m)	
				1.43 (m)	
OCH_2_CH_2_CH_2_CH_3_-20			13.7	1.87 (t, 7.2)	

**Table 2:** In vitro antiplasmodial and Larvicidal activities of compounds **1**–**2**.

Compounds	IC_50_ (μM) ^a^	LC_50_ (μM) ^b^
**1**	3.60 ± 1.2	60.2 ± 2.3
**2**	25.1 ± 1.3	262.0 ± 8.7
**Chloroquine ^c^**	0.19 ± 0.05	38.6 ± 2.1

^a^ IC_50_ = inhibitory concentration 50%; ^b^ LC_50_ = lethal concentration 50%. Values are means ± SD of triplicate experiments. ^c^ Positive control substance.

## References

[1] Consolacion Y., Ragasa J.G.H., John A.R. (2002). New Furanoid Diterpenes from *Caesalpinia pulcherrima*. J. Nat. Prod..

[2] Ma G.X., Yuan J.Q., Wu H.F., Cao L., Zhang X.P., Xu L.J., Wei H., Wu L.Z., Zheng Q.X., Li L.Y. (2013). Caesalpins A–H, Bioactive Cassane-Type Diterpenes from the Seeds of *Caesalpinia minax*. J. Nat. Prod..

[3] Ma G.X., Wu H.F., Chen D.L., Zhu N.L., Zhu Y.D., Li P.F., Sun Z.H., Yang J.S., Xu X.D. (2015). Antimalarial and Antiproliferative Cassane Diterpenes of *Caesalpinia sappan*. J. Nat. Prod..

[4] Yodsaoue O., Cheenpracha S., Karalai C., Ponglimanont C., Chantrapromma S., Fun H.K., Kanjana O.A. (2008). Phanginin A–K, diterpenoids from seeds of *Caesalpinia sappan* Linn. Phytochemistry.

[5] Pudhom K., Sommit D., Suwankitti N., Petsom A. (2007). Cassane Furano-diterpenoids from the Seed Kernels of *Caesalpinia bonduc* from Thailand. J. Nat. Prod..

[6] Dickson R.A., Houghton P.J., Hylands P.J. (2007). Antibacterial and antioxidant cassanediterpenoids from *Caesalpinia benthamiana*. Phytochemistry.

[7] Cheenpracha S., Karalai C., Ponglimanont C., Chantrapromma K., Laphookhieo S. (2006). Cassane-type diterpenes from the seeds of *Caesalpinia crista*. Helv. Chim. Acta.

[8] Xu X.D., Yang J.Q., Zhou X.Y., Li W.P., Zhu N.L., Wu H.F., Li P.F., Sun Z.H., Yang J.S., Ma G.X. (2016). Cassane diterpenes with oxygen bridge from the seeds of *Caesalpinia sappan*. Fitoterapia.

[9] Wang W.X., Xiong J., Tang Y., Zhu J.J., Li M., Zhao Y., Yang G.X., Xia G., Hu J.F. (2013). Rearranged abietane diterpenoids from the roots of *Clerodendrum trichotomum* and their cytotoxicities against human tumor cells. Phytochemistry.

[10] Rayanil K., Limpanawisut S., Tuntiwachwuttikul P. (2013). Entpimarane and enttrachylobane diterpenoids from *Mitrephora alba* and their cytotoxicity against three human cancer cell lines. Phytochemistry.

[11] Nguyen H.X., Nguyen N.T., Dang P.H., Ho P.T., Nguyen M.T.T., Can M.V., Dibwe D.F., Ueda J.Y., Awale S. (2016). Cassane ditrpenes from the seed kernels of *Caesalpinia sappan*. Phytochemistry.

[12] Zhang J.Y., Abdel-Mageed W.M., Liu M.M. (2013). Caesanines A-D, New Cassane Diterpenes with Unprecedented N Bridge from *Caesalpinia sappan*. Org. Lett..

[13] Desjardins R.E., Canfield C.J., Haynes J.D., Chulay J.D. (1979). Quantitative assessment of antimalarial activity in vitro by a semiautomated microdilution technique. Antimicrob. Agents Chemother..

[14] Trager W., Jensen J.B. (1976). Human Malaria in Continuous Culture. Science.

[15] Vennerstrom J.L., Arbe-Barnes S., Brun R., Charman S.A., Chiu F.C.K., Chollet J., Dong Y., Dorn A., Hunziker D., Matile H. (2004). Identification of an antimalarial synthetic trioxolane drug development candidate. Nature.

[16] Kovendan K., Murugan K., Panneerselvam C., Aarthi N., Mahesh P.K., Subramaniam J., Amerasan D., Kalimuthu K., Vincent S. (2012). Antimalarial activity of *Carica papaya* (Family: Caricaceae) leaf extract against *Plasmodium falciparum*. Asian Pac. J. Trop. Dis..

[17] Abbott W.S. (1925). A method of computing the effectiveness of insecticides. J. Econ. Entomol..

[18] Finney D.J. (1971). Probit Analysis.

